# Effects of diet education on empowerment for individuals who have an increased risk of developing breast or colon cancer: A pilot study

**DOI:** 10.1002/jgc4.1584

**Published:** 2022-05-03

**Authors:** Kaitlyn Tlusty, Mariah Jackson, Bronson Riley, Terri Blase

**Affiliations:** ^1^ Division of Genetic Counseling Education College of Allied Health Professions University of Nebraska Medical Center Omaha Nebraska USA; ^2^ Division of Medical Nutrition Education College of Allied Health Professions University of Nebraska Medical Center Omaha Nebraska USA; ^3^ Southeast Nebraska Cancer Center Lincoln Nebraska USA; ^4^ Munroe‐Meyer Institute for Genetics and Rehabilitation University of Nebraska Medical Center Omaha Nebraska USA

**Keywords:** education, genetic counseling, health promotion

## Abstract

Strong evidence indicates following a healthy diet reduces cancer risk; however, the impact of diet education on empowerment on individuals with an increased cancer risk has not been evaluated. Study participants included patients who had met with a cancer genetic counselor without a history of cancer. Participants received pre‐ and post‐diet education surveys including questions to measure empowerment and feedback for diet education in relation to cancer risk. Empowerment was measured using a ten‐question survey adapted from the Genetic Counseling Outcome Scale. The diet education intervention consisted of viewing an infographic created for this study based on recommendations for diets that reduce cancer risk by the World Cancer Research Fund and American Institute for Cancer Research. Twenty‐eight participants completed both surveys and reviewed the diet education intervention. There was no change in empowerment between pre‐ and post‐ diet education (mean change = −0.5; *p* = 0.49). Participants previously learned about the relationship between a healthy diet and cancer risk reduction from several sources including family and friends (25.0%), online (25.0%), and primary care providers (25.0%). Most participants preferred diet education to be delivered online (42.9%), followed by on paper (39.3%), and in‐person delivery (17.9%). This pilot study promotes further investigation on the impact of diet or lifestyle education on individuals who have a predisposition to developing cancer. While the results demonstrated no change in empowerment because of diet education, the results established a desire for learning about a healthy diet related to cancer risk and preferences for the modes of delivering education.

**What is known about this topic?**

Individuals who have an increased risk for developing cancer may feel powerless about their risk. While there is evidence that a healthy diet reduces cancer risk, this is not a topic that is usually discussed with individuals who have increased cancer risk during genetic counseling appointments.

**What this paper adds to the topic?**

This research study served as a pilot study for examining the effects of diet education on empowerment for individuals who have an increased risk for developing cancer. This study identified a desire for learning about the relationship between a healthy diet and cancer risk reduction, and it provided insight about preferences for learning diet information.

## 1 INTRODUCTION

Individuals who have an increased risk of developing cancer because of family history or a known pathogenic genetic variant may have increased levels of anxiety, hopelessness, and feel like they are not in control of their situation (Bennett et al., 2008; Lodder et al., 2001; Trask et al., 2001). These individuals are often referred to a genetic counselor and are provided with recommendations for cancer screening and risk reduction based on discussions with their healthcare team and published guidelines from the National Comprehensive Cancer Network (NCCN). The NCCN guidelines include recommendations regarding enhanced cancer surveillance, risk‐reducing surgical options, and healthy lifestyle recommendations to reduce cancer risk. While healthy lifestyle recommendations are included in the guidelines, genetic counselors do not typically talk about these recommendations with their patients (Albada et al., 2014). For a patient population that may not have control over their genetic risk, lifestyles (especially diet) can be controlled and modified. Patient outcomes of receiving information about lifestyle recommendations have not been examined in a cancer genetic counseling population, even though this is hypothesized to empower these individuals or help them feel a sense of control.

### 1.1 Relationship between diet and cancer

There is a known relationship between healthy diet patterns and cancer risk reduction (King et al., 2003; Ko et al., 2013), with specific risk reduction for breast and colon cancer when following lifestyle recommendations made by the American Cancer Society (ACS), American Institute for Cancer Research (AICR), and the World Cancer Research Fund (WCRF) (Kohler et al., 2016; Petimar et al., 2019; Solans et al., 2020; Turati et al., 2020). Not only do healthy lifestyles reduce cancer risk in the general population, but also healthy diets (rich in fruits, vegetables, whole grains, and healthy protein sources) have been found to reduce the cancer risk for individuals with a genetic predisposition for developing cancer. For example, individuals with pathogenic BRCA genetic variants who had a healthy diet were less likely to develop breast cancer than those with an unhealthy diet (Nkondjock & Ghadirian, 2007).

### 1.2 Diet education within genetic counseling

Because of the evidence for cancer risk reduction, it may be possible to give individuals who have an increased risk for developing cancer a sense of control by educating them about these lifestyle modifications. The research regarding genetic risk and lifestyle education is limited, especially in populations of individuals with a genetic predisposition to cancer. Furthermore, the existing body of evidence, primarily in multifactorial diseases, is conflicting. Some studies have revealed that individuals change their behaviors and lifestyle after learning they have a genetic predisposition to a multifactorial disease. For example, Takeshima et al. (2013) demonstrated that individuals were likely to reduce their salt intake after learning about their genetic predisposition to salt‐sensitive hypertension. Conversely, the study by Godino et al. (2016) revealed no change to behavior after receiving genetic risk information about developing Type II diabetes. Additionally, there have been conflicting data on the impact of disclosure of genetic risk information of cardiovascular disease. Hypotheses by some groups suggest the disclosure of genetic risk information related to cardiovascular disease may motivate behavioral change, while others still fail to find significant impact (Bennett et al., 2008; Hietaranta‐Luoma et al., 2015; Khera et al., 2017). While research has been contradictory, hypotheses by some groups suggest the disclosure of genetic risk information related to cardiovascular disease may motivate behavioral change (Bennett et al., 2008; Hietaranta‐Luoma et al., 2015; Khera et al., 2017). While behavior change has been studied for some multifactorial diseases, there is a gap in the literature on genetic predispositions to cancer. Past research on genetic risk and lifestyle modifications have examined whether individuals who see cancer genetic counselors are at an optimal weight and follow cancer risk‐reducing lifestyles. For example, the majority of men who see a genetic counselor for inherited prostate cancer risk are overweight or obese and do not consume the recommended amount of fruits, vegetables, or fiber in their diet (Milliron et al., 2019). Therefore, it would be beneficial for individuals to make improvements in their lifestyle to reduce their cancer risk.

While it would be valuable for education about diet and healthy lifestyles within genetic counseling sessions (Bennett et al., 2008; Milliron et al., 2019; Quillin, 2016), a discussion about diet only occurs about 27% of the time (Albada et al., 2014). When education about diet and healthy lifestyles is included in the genetic counseling discussion, the patient is more likely to initiate these conversations than the genetic counselor. Additionally, genetic counselors tend to be vague when describing a healthy lifestyle (Albada et al., 2014). Genetic counselors may lack awareness and confidence in lifestyle modifications even though educating individuals about lifestyle has been shown to be empowering. Therefore, there is an opportunity for genetic counselors to become more aware of diet and exercise habits to further impact a patient's sense of control.

### 1.3 Empowerment

Individuals may be impacted in several different ways if a genetic counselor educates them about modifications they can make in their everyday life. The type of impact may include increasing empowerment and self‐efficacy, as well as decreasing anxiety. Empowerment has been defined by the World Health Organization (WHO) as ‘a process by which people gain control over the factors and decisions that shape their lives’. Because empowerment has been defined as impacting one's life, it has been studied in patients to improve health outcomes. Patient empowerment is positively correlated with following health recommendations and a higher sense of wellbeing (Brown & Bussell, 2011; Ison et al., 2019; Kambhampati et al., 2016).

Even though health empowerment is described as a process that promotes one's health, there are not standardized methods of evaluating empowerment within interventions (Lindacher et al., 2018), and different healthcare specialties have created their own methods for evaluating it. One such method, the Genetic Counseling Outcome Scale (GCOS), is a validated patient‐reported outcome measure of empowerment specifically in the clinical genetics setting (McAllister et al., 2011). The GCOS has been utilized across genetic counseling specialties and has been successful due to its design to analyze change pre‐ to post‐intervention.

### 1.4 Research aims

Previous literature reveals a sense of powerlessness and lack of control in individuals who have an increased risk of developing cancer. One area that can be controlled that reduces cancer risk is following a healthy diet. While individuals can control this factor in cancer risk, genetic counselors do not typically provide diet education. There also remains a gap regarding the impact of diet education for individuals who have increased cancer risk. Therefore, there is an opportunity for genetic counselors to provide diet education to patients who have an increased risk of developing cancer which may also have the potential for empowerment and giving control back to this patient population. Genetic counselors have the unique opportunity to individualize patient education by incorporating diet modifications guided by their knowledge of personal medical history, family history, and genetic testing which may further empower individuals to take action in their health. Therefore, this pilot study aims to determine whether individuals are empowered by reviewing a diet education infographic when they have an increased risk for developing breast or colon cancer.

## 2 METHODS

### 2.1 Participants

Individuals were invited to participate in the study if they attended an appointment (of any delivery model) with a cancer genetic counselor to discuss their risk for breast or colon cancer, were nineteen years of age or older, and did not have a personal history of cancer. The indications for seeing a genetic counselor included having a family history of breast or colon cancer as this may increase the risk for cancer development (Akhtar et al., 2008; Monticciolo et al., 2018) and carrying pathogenic genetic variants related to hereditary cancer. Participants were recruited after genetic counselor appointments at the University of Nebraska Medical Center Hereditary Cancer Clinic or the Southeast Nebraska Cancer Center via flyers directly after their genetic counseling appointments or via mailed advertisements if their genetic counseling appointment was within the last two years. Additionally, participants were recruited via social media posts on hereditary cancer support group webpages.

### 2.2 Instrumentation

#### 2.2.1 Surveys

Two surveys, a 19‐question pre‐diet education survey and a 23‐question post‐diet education survey, were designed by the study team (see Supplemental file). The response options in both surveys included multiple‐choice (select one; select all that apply), open‐ended, and seven‐point Likert scale responses. In the pre‐diet education survey, there were six questions about demographics. In the post‐diet education survey, there were three Likert‐response questions about ability and likelihood to alter diet, three Likert‐response questions about understanding of the diet education infographic, and four multiple‐choice questions regarding preferences for receiving diet information.

In both the pre‐ and post‐diet education surveys, there were thirteen questions repeated to compare responses. Three of the thirteen repeated questions were related to the understanding of cancer risk and likelihood to follow through with screening recommendations and diet changes. Ten of the thirteen repeated questions were used to measure empowerment. The empowerment questions were adapted from the Genetic Counseling Outcome Scale (GCOS) by McAllister et al. (2011) and tailored to this study and specific to the study goals.

The GCOS is a 24‐question patient‐reported outcome measure that is validated to evaluate empowerment in a clinical genetics setting. From the seven‐point Likert scale responses, a higher score indicates a greater level of empowerment. Some of the responses are reverse scored due to negative wording of the questions. With the modified GCOS in this study using ten questions, the possible scores ranged from 10 (least empowered) to 70 (most empowered). To maintain a similar measure of empowerment to the 24‐question GCOS, the ten questions selected for this study had high loading values and were distributed across five of the communalities from McAllister et al. (2011).

#### 2.2.2 Diet education infographic

The diet education infographic was created utilizing the AICR/WCRF diet recommendations for reducing cancer risk (Supplementary Information). The American Institute for Cancer Research (AICR) is a reputable research organization, and their guidelines are used by dietitians and physicians in the United States. The AICR, in conjunction with the WCRF, have developed specific cancer prevention recommendations based on a comprehensive analysis, from their Third Expert Report (Shams‐White et al., 2019). A standardized scoring system has been established for these guidelines and adherence to these guidelines has been studied with evidence of decreased risk for breast and colorectal cancers, among others (Petimar et al., 2019; Shams‐White et al., 2019; Turati et al., 2020). Therefore, these recommendations, having a strong scientific basis in cancer prevention and being applicable to the American diet, were an appropriate choice to use as the basis for the diet education.

The infographic featured four of the ten AICR/WCRF cancer prevention recommendations, focusing on easily identifiable alternative food and beverage options, listed as ‘simple swaps’. The remaining recommendations, including limiting alcohol consumption, were listed beneath the highlighted recommendations with an online reference for further information. The authors, consisting of genetic counselors and a registered dietitian, collaborated to create the infographic using Microsoft PowerPoint which was converted to a PDF.

### 2.3 Procedures

Eligible participants reviewed a flyer with a link to participate in the online research study. Then, participants were provided with introductory information, consented to the study, and completed a baseline (pre‐diet education) survey through RedCap. One week after completion of the pre‐diet education survey, participants were emailed a link to the second survey. To decrease dropout rates, this link included the diet education infographic along with the follow‐up (post‐diet education) survey through RedCap. The collection of data took place from June 2, 2020, until December 15, 2020. There were no financial incentives for completing the surveys. This study was deemed exempt by the University of Nebraska Medical Center Institutional Review Board (IRB#: 396–20‐EX).

### 2.4 Data analysis

Baseline descriptive statistics were reported for all continuous and categorical variables (mean, standard deviation, counts, and percentages). Differences in baseline and follow‐up values were measured via paired *t*‐tests with a two‐tailed distribution. Change scores for individual questions between pre‐ and post‐diet education were calculated by subtracting the corrected pre‐score from the corrected post‐score for each question. Empowerment questions 2, 5, 6, 9, and 10 were reverse scored. Empowerment scores were only calculated for participants who reviewed the diet education PDF and completed both empowerment scale surveys, while the incomplete responses were omitted. To measure empowerment for each participant, the ten survey questions were summed together after reverse coding appropriate questions for a total empowerment score, which was performed separately for the pre‐ and post‐diet education. The difference in the summed scores was also calculated to show the overall change in the survey response.

The analyses were performed using Microsoft Excel and SAS software version 9.4 (SAS Institute Inc., Cary, NC). A *p*‐value < 0.05 was considered statistically significant when comparing two groups. Reliability of the empowerment scale was measured using a standardized Cronbach's Coefficient Alpha in which a value ≥0.70 was considered acceptable.

## 3 RESULTS

### 3.1 Sample demographics

A total of 49 participants enrolled in the study and completed the first survey. Of those enrolled, 38 participants completed both surveys. Of those who completed both surveys, ten participants recorded that they did not review the diet education PDF. Therefore, only the 28 participants who completed both surveys and reviewed the diet education PDF were reported in the results (Table 1).

**TABLE 1:** Patient demographics

	*N* = completed intervention (%)
Total completed surveys	38
Total reviewed intervention	28 (100)
Gender	
Female	26 (92.9)
Male	2 (7.1)
Ethnicity	
White	26 (92.9)
Hispanic/Latinx	2 (7.1)
Education	
High school	2 (7.1)
Some colleges	5 (17.9)
College graduate or higher	21 (75.0)
Age	
20–29	5 (17.9)
30–39	11 (39.3)
40–49	7 (25.0)
50–59	3 (10.7)
60–69	1 (3.6)
70–79	1 (3.6)
Average	40.1
Range	23–79
Type of cancer—main point of discussion	
Breast	23 (82.1)
Colon	3 (10.7)
Both breast and colon	2 (7.1)
Referral to cancer genetic counselor	
Family history	18 (64.3)
Personal history of gene mutation	2 (7.1)
Both family history and gene mutation	6 (21.4)
None of these	2 (7.1)
Unsure	0

For the 28 participants who completed the diet education intervention, 26 participants (92.9%) identified as female while two participants identified as male. Twenty‐six participants identified as White (92.9%), while two participants identified as Hispanic/Latinx. Participants were between the ages of 23 and 79 years old with an average age of 40.1 years. Most of the population (21/28; 75.0%) completed higher education while five participants (17.9%) had some college education (Table 1).

Participants were asked to provide the reason they were referred to a genetic counselor and the type of cancer that was the main point of discussion during their genetic counseling appointment. Most participants (18/28; 64.3%) were referred based on family history, while two participants (7.1%) were referred because they have a pathogenic genetic variant known to increase the risk of developing cancer. In addition, six participants (21.4%) were referred for both family history and pathogenic genetic variant, and two participants (7.1%) were referred for none of the options provided. No participants selected ‘unsure’ as the reason for referral. Breast cancer (23/28; 82.1%) was the most common cancer discussed with a genetic counselor. In addition, colon cancer was discussed for three participants (10.7%), and both breast and colon were discussed for two participants (7.1%) (Table 1).

### 3.2 Empowerment measure

The empowerment scale was a reliable measure (standardized Cronbach's Coefficient Alpha = 0.73). The difference in the empowerment score pre‐ versus post‐diet education was not statistically significant (Figure S1). The mean pre‐diet education empowerment score was 54.8 (median = 54.5; *SD* = 5.8), while the mean post‐diet education empowerment score was 54.3 (median = 53; *SD* = 5.7). There was no statistical difference from pre‐ to post‐diet education (mean = −0.5; *p* = 0.49).

**FIGURE 1:**
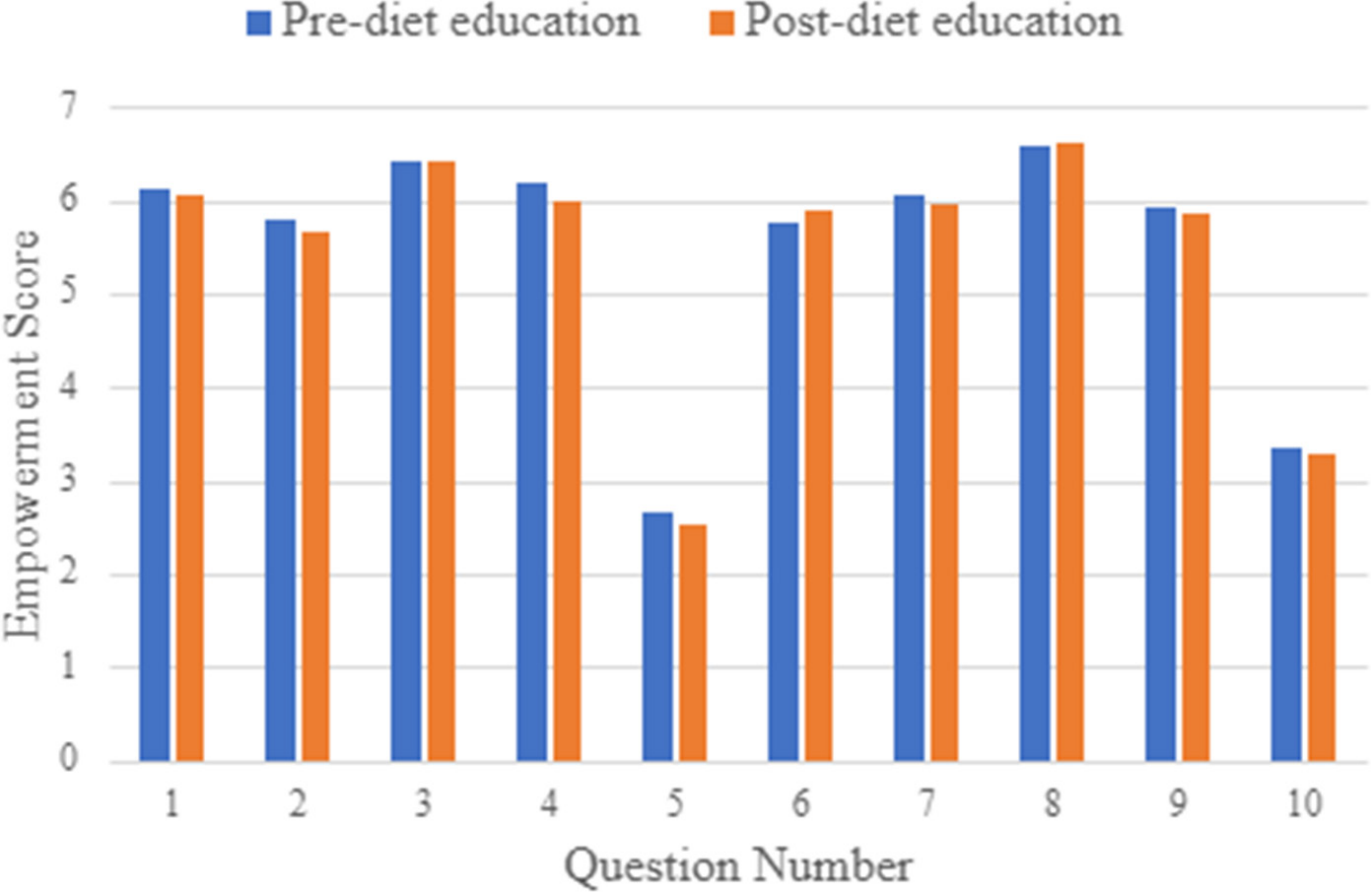
Empowerment scores from the pre‐ and post‐diet education empowerment scale were broken down by question. Questions 2, 5, 6, 9, and 10 were reverse scored (*) to compare empowerment score
I can explain what my risk for cancer means to other people who may need to know.I am powerless to do anything about my risk for cancer *.I understand the reasons why my doctor referred me to the cancer genetics service.I am able to make plans for the future.Having cancer in my family makes me feel anxious *.I don't know what I can do to change how my risk for cancer affects me *.I feel positive about the future.I understand what concerns brought me to the cancer genetics service.I don't know what could be gained from each of the cancer protection and surveillance options available to me *.When I think about cancer in my family, I get upset.

Empowerment items were individually analyzed with possible scores ranging from one to seven (Figure 1). The highest scoring response was question eight (‘I understand what concerns brought me to the cancer genetics service’), which had an average empowerment score of 6.6 pre‐ and post‐diet education. The two lowest‐scoring questions were question five (‘Having cancer in my family makes me feel anxious’) with average empowerment scores of 2.6 pre‐ and 2.5 post‐diet education, and question ten (‘When I think about cancer in my family, I get upset’) with average empowerment scores of 3.4 pre‐ and 3.3 post‐diet education. The other questions related to awareness and understanding, making decisions, changing behavior, and having hope had average scores ranging from 5.5 to 6.4.

### 3.3 Ability and likelihood to alter diet

After receiving diet education, the majority of participants agreed (slightly agreed, agreed, or strongly agreed) that they are capable of changing their diet to live a healthy life (26/28; 92.9%), they will follow recommendations to improve their diet (26/28; 92.9%), and they have all the information they need to improve their diet (21/28; 75.0%) (Figure S2). There were no differences reported to this data because these questions were only asked after receiving the diet education.

### 3.4 Diet education

Of the 28 participants who reviewed the diet education infographic, eleven participants (39.3%) reviewed it 0 to <2 min, fourteen participants (50.0%) reviewed it for 2 to <5 min, and three participants (10.7%) reviewed it for 5 to <10 min (Figure S3). No one reviewed the diet education infographic for more than ten minutes.

All but one participant knew there was an association between diet and cancer risk pre‐diet education (slightly agree = 9/28; strongly agree = 18/28) (Figure S4). All participants understood there may be an association between one's diet and risk for cancer after receiving the diet education (slightly agree = 13/28; strongly agree = 15/28). Next, seventeen participants (60.7%) did not think the diet education was enough information (not at all enough = 6; not quite enough = 11), but eleven participants (39.3%) thought the amount of information was just right (Figure S4). Also, most of the participants thought the diet education was helpful (slightly helpful = 18; extremely helpful = 4).

Diet education was mostly preferred to be received online (12/28; 42.9%) and on paper (11/28; 39.3%), while only some participants preferred to receive diet education in‐person (5/28; 17.9%). Participants were able to leave optional comments at the end of the second survey. Some participants included positive comments including excitement and appreciation about studying this topic. Nobody left negative comments about the diet education.

### 3.5 Sources for diet education

Participants described a variety of sources that they have received information about the association between a healthy diet and cancer risk reduction. A total of fifteen participants (53.6%) reported zero or one source and thirteen participants (46.4%) reported two, three, or four sources about where they learned this information. A majority of these sources were from a primary care provider (7/28; 25.0%), a family or friend (7/28; 25.0%), online (7/28; 25.0%), and from an oncologist (5/28; 17.9%). A total of six participants (21.4%) listed other sources of learning about this risk including research, medical publication, and one participant identified themself as a dietitian.

## 4 DISCUSSION

There are data that demonstrate that individuals who have increased cancer risk may feel increased anxiety and a sense of powerlessness (Bennett et al., 2008; Lodder et al., 2001; Trask et al., 2001), and there is a gap in literature for whether diet education would be a way to empower this patient population. In this pilot study, individuals at risk for cancer who had previously met with a cancer genetic counselor did not have a change in the empowerment measure or likelihood to alter their diet as a result of a diet education infographic. This sample had the highest empowerment scores on items addressing hope, cognitive awareness, and ability to change behavior, and the lowest empowerment scores on items addressing their emotions relating to family members with a diagnosis of cancer. Additionally, this sample showed interest in receiving diet education related to reducing cancer risk, and they had a variety of preferences for receiving this information.

### 4.1 Empowerment

Previous research has examined the effectiveness of using genetic risk information to promote lifestyle and diet changes and revealed no change in behavior or conflicting evidence for whether behavior is changed (Godino et al., 2016). The results of this study revealed no change in empowerment score after diet education for individuals who had previously met with a genetic counselor. While our study and previous studies are conceptually different, the explanation for the lack of effectiveness may overlap. Genetic risk information in combination with diet or lifestyle interventions may not be an effective way to incite a sense of empowerment for patients or drive them to change.

While acknowledging the overlap with the lack of change in previous studies, we also hypothesize the reason that few participants increased their empowerment scores was because of the high empowerment scores and awareness of the healthy diet relationship at baseline. The vast majority of the participants in the study were White, middle‐aged women who have received higher education. Most participants began the study with a high empowerment score and were already aware of the relationship between a healthy diet and cancer risk reduction. Thus, the diet education infographic may not have shown effects in empowerment due to not providing enough information relative to the high knowledge base of the participants, as they acknowledged the information in the diet education infographic was insufficient. Due to recruitment from online support groups, the participants may have already been empowered and performed their own research on available educational and support resources. Similarly, Hu et al. (2012) described details of online health support group participants prior to medical appointments and found they were most likely to use online health resources if they felt a sense of control, experienced negative emotions because of the illness, and had higher literacy related to online health information.

While overall empowerment was already high and without change from the empowerment scale, participants had common responses to the ten individual survey questions which revealed areas of higher and lower empowerment. They were most empowered with having hope, cognitive awareness, and ability to change behavior. They were least empowered about emotions relating to family members who have had a diagnosis of cancer. This finding suggests having family members with a diagnosis of cancer may cause negative emotions for an individual despite how much information they know about their risk and screening options, and it provides an opportunity for genetic counselors to help their patients.

### 4.2 Resources and health empowerment

Participants had access to many different resources including family, friends, healthcare providers, and research publications. Social resources have been identified to stimulate health empowerment (Shearer, 2009), which provides further evidence into the participants’ level of empowerment. This finding promotes expanding access to resources to aid in empowerment.

### 4.3 Delivery of diet education

The majority of participants preferred diet education to be delivered online or on paper. A smaller proportion of participants favored in‐person education delivery. The variation in the delivery preferences may be associated with different learning styles. When information is tailored to an individual's learning style, they have improved understanding, and in turn may become more empowered (Giuse et al., 2012). Other factors besides learning style may contribute to empowerment, such as engagement, which was lacking in this pilot study. Specifically, participants only viewed an infographic, but they did not interact with it. Fewer participants noted ‘strongly agree’ that there is an association between diet and cancer risk post‐diet education compared with pre‐diet education. While these findings are unlikely to be significant, it is possible that fewer participants ‘strongly agreed’ because of the type of intervention and the lack of engagement with the diet education.

Of course, there are opportunities to tailor to learning style while making diet education more engaging. Although the preference for receiving diet education in‐person was the least selected option for this study population, an in‐person approach may actually provide more engagement and be more likely to lead to change compared with online or on paper. Specifically, motivational interviewing is a counseling approach that allows the patient to explore internal motivation to cause change. It has been shown to stimulate empowerment and engagement, and it has been shown to lead to changes in lifestyle behaviors (Barrett et al., 2018; McCarley, 2009; Resnicow et al., 2015). Genetic counselors can utilize techniques in motivational interviewing during a genetic counseling session to empower patients and invoke change. The combination of an interactive approach such as motivational interviewing along with a paper or online education tool such as the one created for this study may even cause additive effects on empowerment and be more likely to cause change.

### 4.4 Interest in diet education

Participants were interested and excited about learning about the relationship between a healthy diet and cancer risk reduction, which reveals a potential benefit of including this information within the hereditary cancer community. While the diet education intervention was reported to not be enough information, it was reported to be helpful. In the optional comments section, participants reported they rely on their own online research and are unaware of which sources are the most credible. The drive to learn about nutrition recommendations from the participants in the study may indicate a desire for talking about a healthy diet outside of a dietitian's office, and this information can be paired with genetic risk.

## 5 LIMITATIONS

The study had a small sample size that lacked diversity. The majority of the participants were of similar demographics: middle‐aged White women with college education. Future studies may recruit from community clinics to incorporate individuals of all backgrounds to improve generalizability. There were also some technical issues because several individuals left comments that they were unable to open the diet education infographic in their internet browser.

Additionally, the empowerment scale was adapted from the GCOS‐24 for this study and has not been validated on its own as a measure of empowerment. Other variables and patient characteristics could be evaluated to determine the impact of diet education on empowerment for individuals who have an increased risk for developing cancer. Of note, while empowerment is described as a process that promotes one's health, a different outcome may be more ideal to better understand the impact of diet education for this population. For example, physical measurements of weight, dietary intake journals or questionnaires, and the transtheoretical model have been utilized to evaluate the effectiveness of diet interventions (Armitage, 2010; Spahn et al., 2010; Spencer et al., 2007). In addition to changing the outcome, this study focused on diet changes only instead of all lifestyle changes that could impact cancer risk including information about exercise, tobacco use, and alcohol consumption.

The data were collected during the COVID‐19 pandemic. It is possible that this may have impacted the results. For example, patients may have met with their genetic counselor while utilizing various service delivery models. The pandemic may have also affected the participation in online support groups, in which most participants were recruited from.

## 6 PRACTICE IMPLICATIONS

Empowerment was unchanged as a result of the diet education from this study, but participants were interested in learning about diet related to cancer risk and they found this information helpful. Genetic counselors can help patients by having resources available for patients who may find nutrition information valuable. While there are currently no guidelines available to make referrals for this population to meet with medical nutritionists or dietitians, a referral base can be established for the appropriate professionals. Additionally, participants were least empowered for questions related to having cancer in the family. Genetic counselors can continue to highlight the importance of taking a family history to build rapport, perform a psychosocial assessment, and provide counseling for patients. Lastly, this study added to previous research showing that individuals who have many resources available to them are more likely to be empowered. Healthcare providers can provide education during community outreach, have credible resources readily available, or encourage patients to join health‐related support groups to promote their patient population's perception of resources available to them.

## 7 RESEARCH RECOMMENDATIONS

This pilot study may be replicated and adapted for a larger‐scale study. A different outcome such as dietary intake questionnaires or stage of change may be considered for future work. Participants were interested in learning about diet education, but the diet education did not have an impact on empowerment. The diet education for this study was provided as a brief online PDF, but a more engaging diet education intervention may be beneficial. For example, motivational interviewing has been proven to be beneficial to empower individuals and cause changes in lifestyle behaviors. Future research can provide instructions or even training on motivational interviewing directed for genetic counselors to use during a genetic counseling session. As another example, participants may not be motivated by lifestyle changes because of the level of genetic risk; however, a future study could compare the level of genetic risk to determine the effects on empowerment as a result of diet education.

## 8 CONCLUSION

This research study serves as a pilot study that can easily be replicated for future research around the impact to empowerment by diet education for individuals who have an increased risk for developing cancer. While the diet education used in our study did not impact empowerment on a highly empowered group of participants, the interest in diet education from individuals who have seen a genetic counselor is promising. Individuals described different sources of receiving health‐related information and preferences for receiving diet education which reveals a desire for healthcare providers to tailor delivery of information to the individual. Future studies can further determine whether combined lifestyle education and genetic risk information is empowering or motivating to patients.

## AUTHOR CONTRIBUTIONS

Kaitlyn Tlusty designed study goals and methods, performed data collection and analysis, wrote and revised the manuscript, and confirms she had full access to all the data in the study and takes responsibility for the integrity of the data and the accuracy of the data analysis. Mariah Jackson, Bronson Riley, and Terri Blase provided oversight and contributed to the study design, data collection, data interpretation, and review of the manuscript. All of the authors gave final approval of this version to be published and agreed to be accountable for all aspects of the work in ensuring that questions related to the accuracy or integrity of any part of the work are appropriately investigated and resolved.

## COMPLIANCE WITH ETHICAL STANDARDS

### Conflicts of interest

Kaitlyn Tlusty, Mariah Jackson, Bronson Riley, and Terri Blase declare they have no conflicts of interest.

### Human studies and informed consent

This study was reviewed and granted an exemption by the University of Nebraska Medical Center Institution Review Board (IRB#: 396‐20‐EX). All procedures followed were in accordance with the ethical standards of the responsible committee on human experimentation (institutional and national) and with the Helsinki Declaration of 1975, as revised in 2000. An introductory paragraph for the first survey served as informed consent. Following the introductory paragraph, individuals voluntarily completed the online survey and submitted their responses.

### Animal studies

No non‐human animal studies were carried out by the authors of this article.

### Data availability statement

The data that support the findings of this study are available from the corresponding author upon reasonable request.

## Supporting information

Fig S1

Fig S2

Fig S3

Fig S4

Supplementary Material

Supplementary Material

## References

[jgc41584-bib-0001] Akhtar, S. , Sinha, S. , McKenzie, S. , Sagar, P. M. , Finan, P. J. , & Burke, D. (2008). Awareness of risk factors amongst first degree relative patients with colorectal cancer. Colorectal Disease, 10(9), 887–890. 10.1111/j.1463-1318.2008.01502.x 18384422

[jgc41584-bib-0002] Albada, A. , Vernooij, M. , van Osch, L. , Pijpe, A. , CanDulmen, S. , & Ausems, M. G. E. M. (2014). Does and should breast cancer genetic counseling include lifestyle advice? Familial Cancer, 13, 35–44. 23934600 10.1007/s10689-013-9672-5

[jgc41584-bib-0003] Armitage, C. J. (2010). Can variables from the transtheoretical model predict dietary change? Journal of Behavioral Medicine, 33(4), 264–273. 10.1007/s10865-010-9261-0 20407921

[jgc41584-bib-0004] Barrett, S. , Begg, S. , O’Halloran, P. , & Kingsley, M. (2018). Integrated motivational interviewing and cognitive behaviour therapy for lifestyle mediators of overweight and obesity in community‐dwelling adults: A systematic review and meta‐analyses. BMC Public Health, 18(1), 1–10. 10.1186/s12889-018-6062-9 PMC6173936 30290793

[jgc41584-bib-0005] Bennett, P. , Wilkinson, C. , Turner, J. , Brain, K. , Edwards, R. T. , Griffith, G. , France, B. , & Gray, J. (2008). Psychological factors associated with emotional responses to receiving genetic risk information. Journal of Genetic Counseling, 17(3), 234–241. 10.1007/s10897-007-9136-x 18259848

[jgc41584-bib-0006] Brown, M. T. , & Bussell, J. K. (2011). Medication adherence: WHO cares? Mayo Clinic Proceedings, 86(4), 304–314. 10.4065/mcp.2010.0575 21389250 PMC3068890

[jgc41584-bib-0007] Giuse, N. B. , Koonce, T. Y. , Storrow, A. B. , Kusnoor, S. V. , & Ye, F. (2012). Using health literacy and learning style preferences to optimize the delivery of health information. Journal of Health Communication, 17(sup3), 122–140. 10.1080/10810730.2012.712610 23030566

[jgc41584-bib-0008] Godino, J. G. , van Sluijs, E. M. , Marteau, T. M. , Sutton, S. , Sharp, S. J. , & Griffin, S. J. (2016). Lifestyle advice combined with personalized estimates of genetic or phenotypic risk of type 2 diabetes, and objectively measured physical activity: A randomized controlled trial. PLoS Medicine, 13(11), e1002185. 10.1371/journal.pmed.1002185 27898672 PMC5127499

[jgc41584-bib-0009] Hietaranta‐Luoma, H. L. , Luomala, H. T. , Puolijoki, H. , & Hopia, A. (2015). Using ApoeE genotyping to promote healthy lifestyles in Finland – Psychological impacts: Randomized controlled trial. Journal of Genetic Counseling, 24, 908–921. 25735442 10.1007/s10897-015-9826-8

[jgc41584-bib-0010] Hu, X. , Bell, R. A. , Kravitz, R. L. , & Orrange, S. (2012). The prepared patient: Information seeking of online support group members before their medical appointments. Journal of Health Communication, 17(8), 960–978. 10.1080/10810730.2011.650828 22574697

[jgc41584-bib-0011] Ison, H. E. , Ware, S. M. , Schwantes‐An, T. H. , Freeze, S. , Elmore, L. , & Spoonamore, K. G. (2019). The impact of cardiovascular genetic counseling on patient empowerment. Journal of Genetic Counseling, 28(3), 570–577. 10.1002/jgc4.1050 30680842

[jgc41584-bib-0012] Kambhampati, S. , Ashvetiya, T. , Stone, N. J. , Blumenthal, R. S. , & Martin, S. S. (2016). Shared decision‐making and patient empowerment in preventive cardiology. Current Cardiology Reports, 18(5), 49–016‐0729‐6. 10.1007/s11886-016-0729-6 27098670

[jgc41584-bib-0013] Khera, A. V. , Emdin, C. A. , Drake, I. , Natarajan, P. , Bick, A. , Cook, N. , Chasman, D. , Baber, U. , Mehran, R. , Rader, D. , Fuster, V. , Boerwinkle, E. , Olle, M. , Orho‐Melander, M. , Ridker, P. , & Kathiresan, S. (2017). Genetic risk, adherence to a healthy lifestyle, and risk of coronary artery disease. Journal of the American College of Cardiology, 69(11S), 2560. 10.1016/S0735-1097(17)35949-1

[jgc41584-bib-0014] King, M. C. , Marks, J. H. , & Mandell, J. B. & New York Breast Cancer Study Group (2003). Breast and ovarian cancer risks due to inherited mutations in BRCA1 and BRCA2. Science, 302(5645), 643–646. 14576434 10.1126/science.1088759

[jgc41584-bib-0015] Ko, K.‐P. , Kim, S.‐W. , Ma, S. H. , Park, B. , Ahn, Y. , Lee, J. W. , Lee, M. H. , Kang, E. , Kim, L. S. , Jung, Y. , Cho, Y. U. , Lee, B. K. , Lin, J. H. , & Park, S. K. (2013). Dietary intake and breast cancer among carriers and noncarriers of BRCA mutations in the Korean Hereditary Breast Cancer Study. The American Journal of Clinical Nutrition, 98(6), 1493–1501. 10.3945/ajcn.112.057760 24153343

[jgc41584-bib-0016] Kohler, L. N. , Garcia, D. O. , Harris, R. B. , Oren, E. , Roe, D. J. , & Jacobs, E. T. (2016). Adherence to diet and physical activity cancer prevention guidelines and cancer outcomes: A systematic review. Cancer Epidemiology, Biomarkers & Prevention, 25(7), 1018–1028. 10.1158/1055-9965.EPI-16-0121 PMC4940193 27340121

[jgc41584-bib-0017] Lindacher, V. , Curbach, J. , Warrelmann, B. , Brandstetter, S. , & Loss, J. (2018). Evaluation of empowerment in health promotion interventions: A systematic review. Evaluation & the Health Professions, 41(3), 351–392. 10.1177/0163278716688065 29172696

[jgc41584-bib-0018] Lodder, L. , Frets, P. G. , Trijsburg, R. W. , Meijers‐Heijboer, E. J. , Klijn, J. G. M. , Duivenvoorden, H. J. , Tibben, A. , Wagner, A. , van der Meer, C. A. , van den Ouweland, A. M. W. , & Niermeijer, M. F. (2001). Psychological impact of receiving a BRCA1/BRCA2 test result. American Journal of Medical Genetics, 98(1), 15–24. 11426450

[jgc41584-bib-0019] McAllister, M. , Wood, A. M. , Dunn, G. , Shiloh, S. , & Todd, C. (2011). The genetic counseling outcome scale: A new patient‐reported outcome measure for clinical genetics services. Clinical Genetics, 79(5), 413–424. 10.1111/j.1399-0004.2011.01636.x 21255005

[jgc41584-bib-0020] McCarley, P. (2009). Patient empowerment and motivational interviewing: Engaging patients to self‐manage their own care. Nephrology Nursing Journal, 36(4), 409. 19715108

[jgc41584-bib-0021] Milliron, B. , Bruneau, M. , Obeid, E. , Gross, L. , Bealin, L. , Smaltz, C. , & Giri, V. N. (2019). Diet assessment among men undergoing genetic counseling and genetic testing for inherited prostate cancer: Exploring a teachable moment to support diet intervention. The Prostate, 79, 778–783. 10.1002/pros.23783 30905089 PMC8283914

[jgc41584-bib-0022] Monticciolo, D. L. , Newell, M. S. , Moy, L. , Niell, B. , Monsees, B. , & Sickles, E. A. (2018). Breast cancer screening in women at higher‐than‐average risk: Recommendations from the ACR. Journal of the American College of Radiology, 15(3), 408–414. 29371086 10.1016/j.jacr.2017.11.034

[jgc41584-bib-0023] Nkondjock, A. , & Ghadirian, P. (2007). Diet quality and BRCA‐associated breast cancer risk. Breast Cancer Research and Treatment, 103(3), 361–369. 10.1007/s10549-006-9371-0 17063275

[jgc41584-bib-0024] Petimar, J. , Smith‐Warner, S. A. , Rosner, B. , Chan, A. T. , Giovannucci, E. L. , & Tabung, F. K. (2019). Adherence to the World Cancer Research Fund/American Institute for Cancer Research 2018 recommendations for cancer prevention and risk of colorectal cancer. Cancer Epidemiology and Prevention Biomarkers, 28(9), 1469–1479. 10.1158/1055-9965.EPI-19-0165 PMC6726499 31235471

[jgc41584-bib-0025] Quillin, J. M. (2016). Lifestyle risk factors among people who have had cancer genetic testing. Journal of Genetic Counseling, 25, 957–964. 10.1007/s10897-015-9925-6 26659117

[jgc41584-bib-0026] Resnicow, K. , McMaster, F. , Bocian, A. , Harris, D. , Zhou, Y. , Snetselaar, L. , Schwartz, R. , Myers, E. , Gotlieb, J. , Foster, J. , Hollinger, D. , Smith, K. , Woolford, S. , Mueller, D. , & Wasserman, R. C. (2015). Motivational interviewing and dietary counseling for obesity in primary care: An RCT. Pediatrics, 135(4), 649–657. 10.1542/peds.2014-1880 25825539 PMC4379459

[jgc41584-bib-0027] Shams‐White, M. M. , Brockton, N. T. , Mitrou, P. , Romaguera, D. , Brown, S. , Bender, A. , Kahle, L. L. , & Reedy, J. (2019). Operationalizing the 2018 World Cancer Research Fund/American Institute for Cancer Research (WCRF/AICR) cancer prevention recommendations: A standardized scoring system. Nutrients, 11(7), 1572. 10.3390/nu11071572 31336836 PMC6682977

[jgc41584-bib-0028] Shearer, N. B. C. (2009). Health empowerment theory as a guide for practice. Geriatric Nursing, 30(2 Suppl), 4. 19345857 10.1016/j.gerinurse.2009.02.003 PMC2873187

[jgc41584-bib-0034] Solans, M. , Chan, D. S. M. , Mitrou, P. , Norat, T. , & Romaguera, D. (2020). A systematic review and meta‐analysis of the 2007 WCRF/AICR score in relation to cancer‐related health outcomes. Annals of Oncology, 31(3), 352–368. 32067678 10.1016/j.annonc.2020.01.001

[jgc41584-bib-0029] Spahn, J. M. , Reeves, R. S. , Keim, K. S. , Laquatra, I. , Kellogg, M. , Jortberg, B. , & Clark, N. A. (2010). State of the evidence regarding behavior change theories and strategies in nutrition counseling to facilitate health and food behavior change. Journal of the American Dietetic Association, 110(6), 879–891. 10.1016/j.jada.2010.03.021 20497777

[jgc41584-bib-0030] Spencer, L. , Wharton, C. , Moyle, S. , & Adams, T. (2007). The transtheoretical model as applied to dietary behaviour and outcomes. Nutrition Research Reviews, 20(1), 46–73. 10.1017/S0954422407747881 19079860

[jgc41584-bib-0031] Takeshima, T. , Okayama, M. , Harada, M. , Ae, R. , & Kajii, E. (2013). Effects of disclosing hypothetical genetic test results for salt sensitivity on salt restriction behavior. International Journal of General Medicine, 6, 361. 23696713 10.2147/IJGM.S44979 PMC3658438

[jgc41584-bib-0032] Trask, P. C. , Paterson, A. G. , Wang, C. , Hayasaka, S. , Milliron, K. J. , Blumberg, L. R. , Gonzalez, R. , Murray, S. , & Merajver, S. D. (2001). Cancer‐specific worry interference in women attending a breast and ovarian cancer risk evaluation program: Impact on emotional distress and health functioning. Psycho‐Oncology, 10(5), 349–360. 10.1002/pon.510 11536413

[jgc41584-bib-0033] Turati, F. , Dalmartello, M. , Bravi, F. , Serraino, D. , Augustin, L. , Giacosa, A. , Negri, E. , Levi, F. , & La Vecchia, C. (2020). Adherence to the World Cancer Research Fund/American Institute for Cancer research recommendations and the risk of breast cancer. Nutrients, 12(3), 607. 10.3390/nu12030607 32110887 PMC7146587

