# Supplementary material for: Effects of diet education on empowerment for individuals who have an increased risk of developing breast or colon cancer: A pilot study
Source: J Genet Couns. 2022 May 3;31(5):1138–47. doi: 10.1002/jgc4.1584 (PMC9790378; doi:10.1002/jgc4.1584)
Supplement: Supplementary file 1 — Fig S1 [file JGC4-31-1138-s006.docx]

**
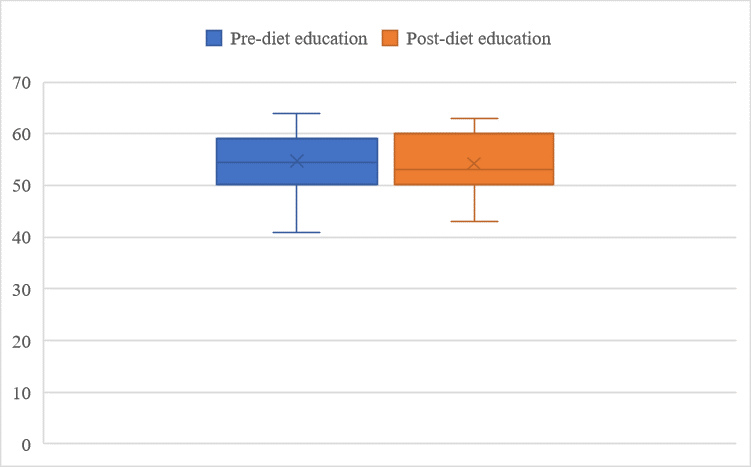
**

**SUPPLEMENTARY FIGURE 1** Total empowerment scores from the pre- and post-diet education empowerment scale. The empowerment scores were corrected and summed for each participant pre-diet education (first survey) and post-diet education (second survey). The range of possible scores was 10-70. The median is displayed by the horizontal bar and the mean is displayed by the “X.”
