# Supplementary material for: Effects of diet education on empowerment for individuals who have an increased risk of developing breast or colon cancer: A pilot study
Source: J Genet Couns. 2022 May 3;31(5):1138–47. doi: 10.1002/jgc4.1584 (PMC9790378; doi:10.1002/jgc4.1584)
Supplement: Supplementary file 2 — Fig S2 [file JGC4-31-1138-s001.docx]

**
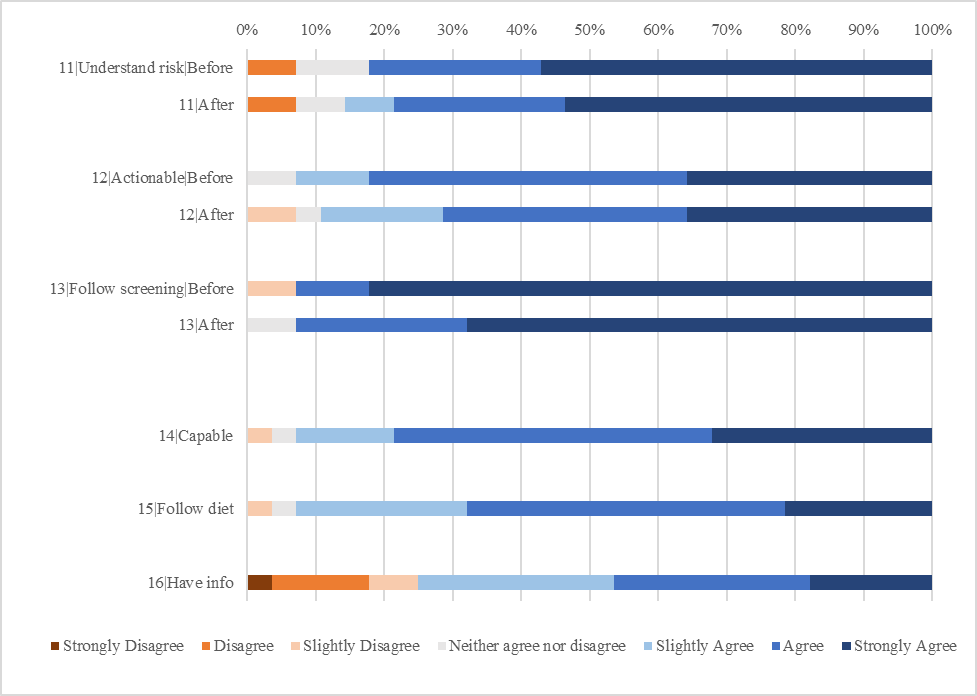
**

**SUPPLEMENTARY FIGURE 2** Likert scale responses broken down into the percent of participants who listed each answer. Questions 11-13 were asked pre- and post-diet education and were relating to cancer risk. Questions 14-16 were asked only post-diet education and were relating to ability and likelihood to alter diet.

11. I understand that my risk for cancer may be increased from the general population.

12. I feel like I can do something actionable about my risk for cancer.

13. I will follow recommendations given to me regarding screening options for my risk for cancer (e.g. mammograms, colonoscopies).

14. I am capable of changing my diet to live a healthy life.

15. I will follow recommendations to improve my diet.

16. I have all of the information I need to improve my diet.
