# Supplementary material for: Effects of diet education on empowerment for individuals who have an increased risk of developing breast or colon cancer: A pilot study
Source: J Genet Couns. 2022 May 3;31(5):1138–47. doi: 10.1002/jgc4.1584 (PMC9790378; doi:10.1002/jgc4.1584)
Supplement: Supplementary file 3 — Fig S3 [file JGC4-31-1138-s003.docx]

**SUPPLEMENTARY FIGURE 3** The amount of time spent reviewing the diet education infographic as a percent of the total participants.
