# Supplementary material for: Effects of diet education on empowerment for individuals who have an increased risk of developing breast or colon cancer: A pilot study
Source: J Genet Couns. 2022 May 3;31(5):1138–47. doi: 10.1002/jgc4.1584 (PMC9790378; doi:10.1002/jgc4.1584)
Supplement: Supplementary file 4 — Fig S4 [file JGC4-31-1138-s002.docx]

**SUPPLEMENTARY FIGURE 4** Understanding of association between diet and cancer risk (17, 18) and diet education feedback (19, 20). Likert scale responses broken down into the percent of participants who listed each answer. Questions were asked post-diet education.

1. Before the diet education, I knew there was an association between diet and cancer risk. Strong - = Strongly disagree; Strong + = strongly agree.
2. After the diet education, I understand there may be an association between one’s diet and cancer risk. Strong – = Strongly disagree; Strong + = strongly agree.
3. The amount of information provided by the diet education infographic was: not at all enough (Strong -), not quite enough, just right, a little too much, or way too much (Strong +).
4. The information about diet from the diet education infographic was: extremely unhelpful (Strong -), slightly unhelpful, neither unhelpful nor unhelpful, slightly helpful, or extremely helpful (Strong +).
