# Supplementary material for: Effects of diet education on empowerment for individuals who have an increased risk of developing breast or colon cancer: A pilot study
Source: J Genet Couns. 2022 May 3;31(5):1138–47. doi: 10.1002/jgc4.1584 (PMC9790378; doi:10.1002/jgc4.1584)
Supplement: Supplementary file 5 — Supplementary Material [file JGC4-31-1138-s004.docx]

**Participation Survey: Page 1**

**Pre diet education survey: Page 2**

**Post diet education survey: Page 5**

**Participation Survey**

Please complete the survey below.

Thank you!

You are invited to take part in a research study. To participate, you must have met with a cancer genetic counselor and be at least 19 years old. There are no known benefits or risks to your participation in this study. It is optional for you to participate. If you choose to participate, you will be asked to complete two online surveys.

1. You will be emailed the link for the first survey.

2. You will be emailed a second survey about one week after you complete your first survey.

3. Each survey will take between 5-10 minutes.

1) What is your email address?

*You will only be contacted for research purposes. __________________________________

Your email address will not be linked with your

answers after you complete the second survey.

**Survey 1**

Please complete the survey below.

Thank you!

You are invited to take part in a research study. To participate, you must have met with a cancer genetic counselor and be at least 19 years old. There are no known benefits or risks to your participation in this study. It is optional for you to participate. If you choose to participate, you will be asked to complete two online surveys.

1. The initial survey will begin after you consent to research.

2. You will be emailed an education tool to view and a second survey about one week from after completing your first survey.

3. Each survey will take between 5-10 minutes.

If you have questions, please email Kaitlyn at Kaitlyn.Tlusty@unmc.edu. Your name or other personal information will not be collected for this study. The email you use will be deleted after you complete the study. You will not receive any emails from the study team after you are done. You will not be able to be identified from your answers in the surveys. You can choose to stop participating at any time.

If you have questions about your rights as a research participant, you may email the UNMC IRB: irbora@unmc.edu.

Principal investigator: Kaitlyn Tlusty; IRB#: 396-20-EX

Institution: UNMC College of Allied Health; 42nd and Emile St, Omaha, NE 68198

If you would like to participate in the study, the first survey will begin when you click Next Page.

2) What is your gender?

Female

Male

Other

Prefer not to answer

3) How would you describe your ethnicity?

White

Hispanic or Latino

Black or African American

Native American or American Indian

Asian or Pacific Islander

Other

Prefer not to answer

4) What is the highest level of education you have completed?

Less than High School

Completed High School

Some college

College graduate or higher

5) What is your age?

__________________________________

6) What type of cancer was the main point of discussion during your genetic counseling session?

Breast cancer

Colon cancer

Both breast and colon cancer

7) What is your understanding of why you were referred to a genetic counselor? (check all that apply)

High risk based on family history of breast or colon cancer

High risk because I have a gene mutation known to increase risk for breast or colon cancer

Both 1 and 2

None of these options

Unsure

**Using the scale below, mark the box next to each statement to indicate how much you agree**

**with the statement. Please answer all the questions. For questions that are not applicable to**

**you, please choose option 4 (neither agree nor disagree).**

**(McAllister et al., 2011)**

- - - Strongly disagree
    - Disagree
    - Slightly disagree
    - Neither disagree nor agree
    - Slightly Agree
    - Agree
    - Strongly agree

8) I can explain what my risk for cancer means to other people who may need to know.

9) I am powerless to do anything about my risk for cancer.

10) I understand the reasons why my doctor referred me to the cancer genetics service.

11) I am able to make plans for the future.

12) Having cancer in my family makes me feel anxious.

13) I don't know what I can do to change how my risk for cancer affects me.

14) I feel positive about the future.

15) I understand what concerns brought me to the cancer genetics service.

16) I don't know what could be gained from each of the cancer protection and surveillance options available to me.

17) When I think about cancer in my family, I get upset.

18) I understand that my risk for cancer may be increased from the general population.

19) I feel like I can do something actionable about my risk for cancer.

20) I will follow recommendations given to me regarding screening options for my risk for cancer (e.g. mammograms, colonoscopies).

This was part of an initial survey. Please look out for an email from us within the next two weeks for the follow-up

survey. Thank you for your responses.

**Survey 2**

Please complete the survey below.

Thank you!

Please view this link to learn about cancer prevention diet recommendations. The link will open in a new tab. To

navigate back, you may click the "back" button or you may return to this tab.

[Attachment: "Cancer Prevention Diet Recommendations.pdf"]

**Using the scale below, mark the box next to each statement to indicate how much you agree**

**with the statement. Please answer all the questions. For questions that are not applicable to**

**you, please choose option 4 (neither agree nor disagree).**

**(McAllister et al., 2011)**

- - - Strongly disagree
    - Disagree
    - Slightly disagree
    - Neither disagree nor agree
    - Slightly Agree
    - Agree
    - Strongly agree

1) I can explain what my risk for cancer means to other people who may need to know.

2) I am powerless to do anything about my risk for cancer.

3) I understand the reasons why my doctor referred me to the cancer genetics service.

4) I am able to make plans for the future.

5) Having cancer in my family makes me feel anxious.

6) I don't know what I can do to change how my risk for cancer affects me.

7) I feel positive about the future.

8) I understand what concerns brought me to the cancer genetics service.

9) I don't know what could be gained from each of the cancer protection and surveillance options available to me.

10) When I think about cancer in my family, I get upset.

11) I understand that my risk for cancer may be increased from the general population.

12) I feel like I can do something actionable about my risk for cancer.

13) I will follow recommendations given to me regarding screening options for my risk for cancer (e.g. mammograms, colonoscopies).

14) I am capable of changing my diet to live a healthy life.

15) I will follow recommendations to improve my diet.

16) I have all of the information I need to improve my diet.

17) We understand many people are busy and may not have the time to spend taking surveys. About how many minutes did you spend reviewing the diet education graphic?

0 minutes (I did not review the diet education graphic)

More than 0 minutes

More than 2 minutes

More than 5 minutes

More than 10 minutes

18) Before the diet education graphic, I knew there was an association between diet and cancer risk.

Strongly disagree

Slightly disagree

Slightly agree

Strongly agree

19) After the diet education graphic, I understand there may be an association between one's diet and risk for cancer.

Strongly disagree

Slightly disagree

Slightly agree

Strongly agree

Not applicable: I did not review the diet education graphic

20) How would you prefer to receive diet information?

In-person

Online

On paper

Other: please specify

21) Please specify how you would prefer to receive diet

information __________________________________

22) From which of the following have you received information about a healthy diet in relation to cancer risk? Check all that apply.

Primary care provider

Nurse

Dietician

Oncologist

Genetic counselor

Online

Family/friend

Other: please list

Not applicable: I have not learned about a healthy

diet in relation to cancer risk

23) Please specify how you have received information about a healthy diet in relation to cancer risk __________________________________

24) How would you describe the amount of information provided about diet?

Not at all enough

Not quite enough

Just right

A little too much

Way too much

25) How helpful was the information on diet?

Extremely unhelpful

Slightly unhelpful

Neither helpful nor unhelpful

Slightly helpful

Extremely helpful

26) You are finished with this survey. Thank you for your

participation in this research program. If you have __________________________________

questions or comments please type below.
