# Supplementary material for: Effects of diet education on empowerment for individuals who have an increased risk of developing breast or colon cancer: A pilot study
Source: J Genet Couns. 2022 May 3;31(5):1138–47. doi: 10.1002/jgc4.1584 (PMC9790378; doi:10.1002/jgc4.1584)
Supplement: Supplementary file 6 — Supplementary Material [file JGC4-31-1138-s005.pdf]

Cancer Prevention Diet Recommendations

Recommendations are from the World Cancer Research Fund & the American Institute for Cancer Research

Research suggests that following these recommendations may reduce your risk for cancer. Any changes you make to your diet from this list may go in some way toward reducing your cancer risk.

World Cancer Research Fund | American Institute for Cancer Research

SIMPLE SWAPS

Eat a diet rich in whole grains, vegetables, fruits, and beans

- Sugary cereal → Steel-cut/rolled oats
- White bread → Whole wheat bread
- White rice or pasta → Brown rice, quinoa
- White potato sides (tater tots, hash browns, fries) → Apples, spinach, black beans

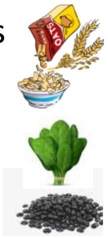

Limit consumption of fast foods and other processed foods high in fat, starches, or sugars

- Fried food or fast food → Baked, steamed, or roasted food
- Butter → Olive oil
- Snack cakes & candy bars → Dark chocolate squares
- Potato chips → Mixed nuts (lightly salted)

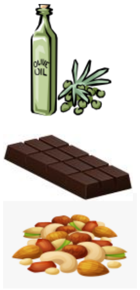

Limit consumption of red and processed meat

- Less: beef & pork → Chicken
- Avoid: bologna & hot dogs → Seafood
- → Tofu

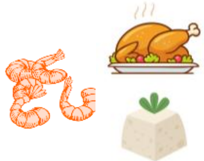

Limit consumption of sugar sweetened drinks

- Pop → Water
- Flavored coffee drinks → Black coffee or unsweetened tea
- Fruit juice → Milk or dairy alternatives (Unsweetened)

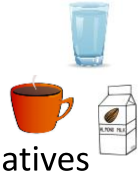

Following these Cancer Prevention Diet Recommendations is likely to:

- Reduce intakes of salt and less healthy fats (saturated and trans)
- Together, these can help prevent other chronic diseases like heart disease or Type 2 diabetes

Other recommendations made by the WCRF and AICR:

- Be a healthy weight
- Be physically active
- Limit alcohol consumption
- Do not use supplements for cancer prevention
- For mothers – breastfeed your baby if you can
- Avoid smoking or other tobacco exposure
- Limit excess sun exposure

For more information go to www.aicr.org.
For more information about the research findings that led to these recommendations, go to https://www.wcrf.org/

University of Nebraska Medical Center
